# The use of a Subjective wellbeing scale as predictor of adherence to neuroleptic treatment to determine poor prognostic factor in African population with Schizophrenia

**DOI:** 10.1192/j.eurpsy.2023.384

**Published:** 2023-07-19

**Authors:** J. J. Boshe, D. Stein, M. Campbell

**Affiliations:** ^1^Mental health and Psychiatry, Kilimanjaro Christian Medical Center, Kilimanjaro, Tanzania, United Republic of; ^2^Mental health and Psychiatry, University of Cape town, Cape town; ^3^Rhodes University, Johannesburg, South Africa

## Abstract

**Introduction:**

Subjective well-being when on neuroleptic treatment (SWBN), has been established as a good predictor of adherence, early response and prognosis in patients with schizophrenia. The 20-item subjective well-being under neuroleptic treatment scale (SWN-K 20) is a self-rating scale that has been validated to measure SWBN. However the SWN-K20 has not been previously used to explore psychosocial and clinical factors influencing a low SWN-K20 score in an African population. This study uses the the SWN-K 20 scale among Xhosa speaking African patients with Schizophrenia to determine factors associated with SWBN in this population

**Objectives:**

To investigate and identify demographic and clinical predictors of subjective well-being in a sample of Xhosa people with schizophrenia on neuroleptic treatment.

**Methods:**

As a part of a large genetic study, 244 study participants with a confirmed diagnosis of schizophrenia completed the translated SWN-K 20 scale. Internal consistency analysis was performed, and convergent analysis and exploratory analysis were conducted using Principal Component Analysis (PCA). Linear regression methods were used to determine predictors of SWBN in the sample population.

**Results:**

When translated into isiXhosa, the sasubscales of SWN-K 20 on their own were observed to be less reliable when compared to the scale in its entirety,internal consistency of 0.86 vs0. 59-0.47. The subscales were therefore noted to be not meaningful in measuring specific constructs but the full scale could be used to determine a single construct of general wellbeing.The validity of the SWN-K20 was further confirmed by moderate correlation scores with Global Assesment functioning scores (GAF), 0.44.There was a significant correlation between overall subjective well-being score with higher education level, and increased illness severity and GAF scores.

**Conclusions:**

Patients’ perception of well-being while on neuroleptic treatment is an essential area of focus when aiming at improving patient centred treatment, compliance and overall treatment outcome. Treating individuals with SMI is difficult and made more complex when patient’s treatment experience and expectations are not elicited. Having a self-reported measurement like the SWN-K 20 available in a validated Xhosa language version provides helpful, possibly broad insights into the subjective well-being experiences of this patient group. Future studies should explore specific symptoms domains that are associated with a change in subjective wellbeing instead of general illness severity.

**Disclosure of Interest:**

None Declared

